# Investigation of the effect of types of two different air filtered full-face masks used in chemical, biological, radiological, nuclear (CBRN) events on endotracheal intubation time: A randomized controlled study

**DOI:** 10.1016/j.heliyon.2024.e28135

**Published:** 2024-03-14

**Authors:** Orhan Delice, Muhammet Özkul

**Affiliations:** Department of Emergency Medicine, Erzurum City Hospital, Erzurum, Turkey

**Keywords:** Chemical, Biological, Radiological, Nuclear (CBRN) events, Endotracheal intubation, Respiratory protective equipment, Air-filtered full-face mask

## Abstract

**Introduction:**

Healthcare personnel may have to intervene with the injured using personal protective equipment depending on the environmental conditions.In injuries occurring in chemical, biological, radiological and nuclear (CBRN) events, healthcare personnel may have to intervene in the injured using personal protective equipment.The equipment used may lead to limitations, especially in cases requiring advanced airway intervention such as intubation. In this study, the effects of personal protective equipment on the intubation times of healthcare personnel were investigated.

**Method:**

This research was planned as a randomized prospective study, and the intubation procedure was performed on a simulation manikin. The intubation times were evaluated among three separate groups, 21 paramedic personnel in each. One group worked without masks, one used front filter masks, and the last worked with side filter masks.

**Results:**

The time spent for intubation by wearing a full-face mask with a side air filter and the intubation times performed without a mask were significantly different (p = 0.011). However, the intubation times of the groups using front and side air-filtered full-face masks were similar (p = 0.279).

**Conclusions:**

Health personnel should use a full-face mask with a front air filter as personal protective equipment during the interventions for the injured who need endotracheal intubation.

## 1 Introduction

As a result of the upper and lower respiratory tracts being affected by chemical, biological, radiological, nuclear (CBRN) events, the injured may need an advanced airway. Especially in mass events, healthcare personnel may have to intervene in CBRN casualties without decontamination procedures being carried out [1]. During the CBRN intervention by the healthcare personnel, the most suitable personal protective equipment (PPE) should be preferred, allowing effective intervention by a limited number of medical personnel in a chaotic environment. The choice of PPEs is made among four levels (A, B, C, D), whose level of protection gradually decreases according to the type and severity of the CBRN event [2]. Level C- PPE usually adequately protects first responders [3]. Full-face mask with air filter from CBRN-PPEs protects healthcare personnel from toxic chemical gases, biological agents (viruses, toxins and bacteria) transmitted through breathing and radiation emitted in the form of particles. However, level A, B and C equipment may restrict sensitive medical procedures. Gloves used as PPEs and full-face masks with air filters may affect the success of fine motor movements such as vascular access or endotracheal intubation [[4], [5], [6], [7]]. Full-face masks from Level C CBRN-PPEs can be binocular, with panoramic visors, front or side air filters [2]. Using the most appropriate PPEs that will not restrict vital interventions during the intervention of CBRN casualties by the healthcare personnel may directly affect survival.

This study aimed to investigate the effect of PPEs (full-face masks with front and side air filters and panoramic visors) used by healthcare professionals on endotracheal intubation time.

## 2 Materials and methods

### 2.1 Paramedic Recruitment

The study was planned as a randomized controlled trial and approved by the Erzurum Medical School local ethics committee (Decision No: 2023/02–15). The participants were 63 paramedic personnel, 30 women and 33 men, with previous human intubation experience and were employed in the Erzurum Province Ambulance Service. After explaining all the study details to the participants, their written informed consent was obtained. The study was planned to be conducted with three groups (A: not using a mask, B: using a front air filter mask, C: using a side air filter mask) with similar participant numbers and gender ratios. The distribution of the participants to the groups was made separately for both genders and by simple random selection. As a result of the selection process, there were 21 first in each group, 10 women and 11 men.

### 2.2 Respiratory protective equipment

**Fig. 1:**
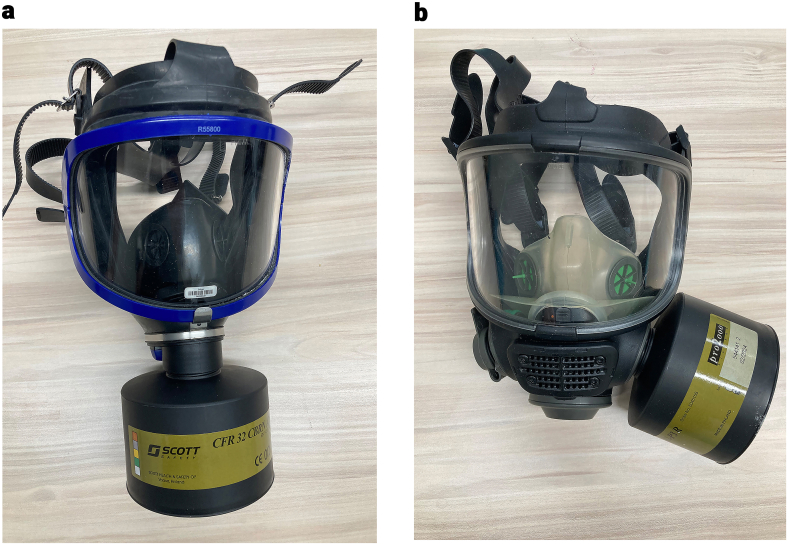
Panoramic full-face mask with front air filter (a) and panoramic full-face mask with side air filter (b).

A panoramic visor X-plore 6300 (Dräger safety AG & Co. KGaA, Luebeck Germany) was used as a front air filter full-face mask (Fig. 1a). A panoramic visor M − 98 (Scott Health & Safety, Skelmersdale, UK) (Fig. 1b) was used for a side air filter full-face mask. CFR 32 CBRN A2B2E2K2–P3 R (Scott Health & Safety, Vaasa, Finland) was used as an air filter in both full-face masks. Both types of face masks were panoramic visor face masks. In both masks, people were given appropriate sizes. These masks were the equipment used in CBRN practical training.

### 2.3 Patient simulator

The study was conducted on a simulation manikin (Advanced Life Support Simulator, Laerdal, Stavanger, Norway) in the Erzurum Ambulance Service training center. The manikin was developed to be performed on intubation, defibrillation and vascular access exercises, and the accuracy of the procedure could be monitored and recorded on the computer screen.

## 3 Study protocol

**Fig. 2:**
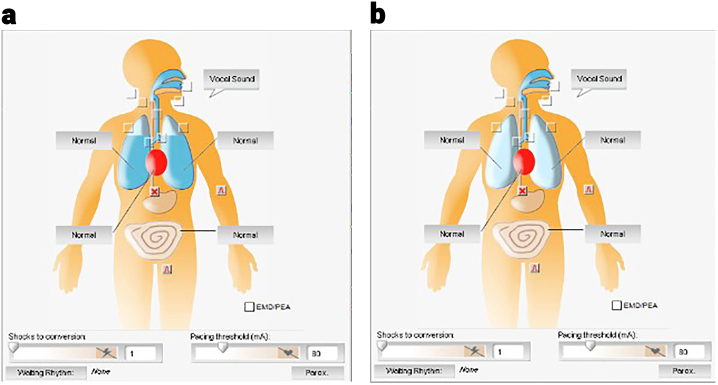
Successful intubation on the manikin (a), unsuccessful intubation on the manikin (b).

First, all groups were given 20 min of endotracheal intubation training separately on a simulation-supported manikin. At the end of the training period, the groups were given 60 min to practice intubation on the manikin, and each participant was asked to perform at least three intubations. After the participants' training was completed, the actual trial phase was started. Group A performed intubation on the manikin without wearing any mask, Group B with front air filter masks and Group C with side air filter masks and panoramic-visors. All participants were kept ready in the hands-free position in front of the simulation manikin, which was laid on the floor just before the intervention. A tracheal tube (ID 7 mm), intubation stylet (10 fr), Macintosh laryngoscope (size 3 blade) and cuff syringe (10 ml) were positioned next to the manikin. Endotracheal intubation was performed with the start command. Intubation was considered complete when the first breath was given. Time was measured in seconds with a stop-watch and recorded. Appropriate intubation was checked from the computer to which the simulation manikin was connected. When the manikin was exhaled for the first time on the computer monitor, the formation of blue color in the lung bases was considered successful (Fig. 2a), and the absence of blue color was regarded as unsuccessful intubation (Fig. 2b).

**Fig. 3:**
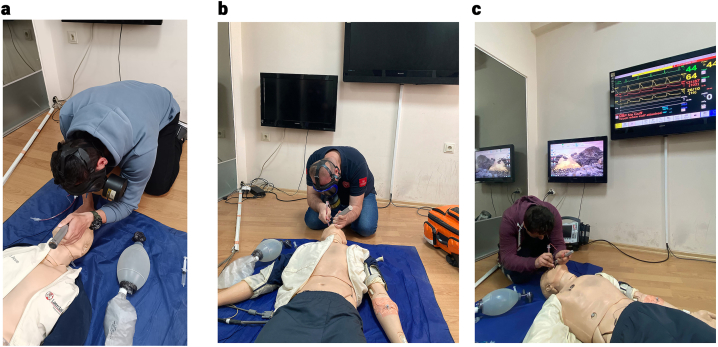
Intubation on the manikin with a panoramic full-face mask with side air filter (a), intubation on the manikin with a panoramic full-face mask with front air filter (b), intubation without a mask (c).

The visuals of the intubation attempt of all three groups on the mannequin are presented in Fig. 3 (a, b, c)

### 3.1 Study hypothesis

H0 There is no difference in intubation times between full-face masks with panoramic visors, front filters and side filters used by healthcare personnel as PPEs.

H1 There is a difference in intubation times between full-face masks with panoramic visors, front filters and side filters, used by healthcare personnel as PPEs.

### 3.2 Statistical analysis

Statistical Package for the Social Sciences (SPSS v26) program was used to analyze the study data. Categorical variables were presented as numbers and percentages, and numerical variables were presented as mean, standard deviation, median, and interquartile range. The suitability of the numerical variables to the normal distribution was evaluated using the Kolmogorov Smirnov Test, z values calculated for skewness and kurtosis, and graphing methods. First, study groups were compared in terms of matching criteria (occupation, age, and duration of professional experience) using chi-square and Kruskal-Wallis tests, respectively. Endotracheal intubation times of the study groups were evaluated with Kruskal-Wallis, and Mann Whitney U tests with Bonferroni correction in post hoc analyses. The statistical significance level was accepted as p < 0.05 in all analyses.

## 4 Results

The study groups consisted of 63 participants, 10 women and 11 men in each group. Age and experience in the profession were similarly distributed in the groups (p = 0.534 and p = 0.476, respectively). The distribution of the participants according to their demographic characteristics and study groups is presented in Table 1.

**Table 1:** Distribution of the participants by demographic characteristics and study groups.

Variables	Groups			*p* value
	Group A	Group B	Group C	
**Age** [(mean age, (SD)]	31.2 (7.4)	29.2 (5.2)	30.6 (8.5)	0.534
**Experience in the profession [**Median years, (IQR)]	10.0 (8.0)	3.0 (11.0)	4.0 (11.0)	0.476

IQR: Inter Quartile Range, SD: Standard Deviation.

**Fig. 4:**
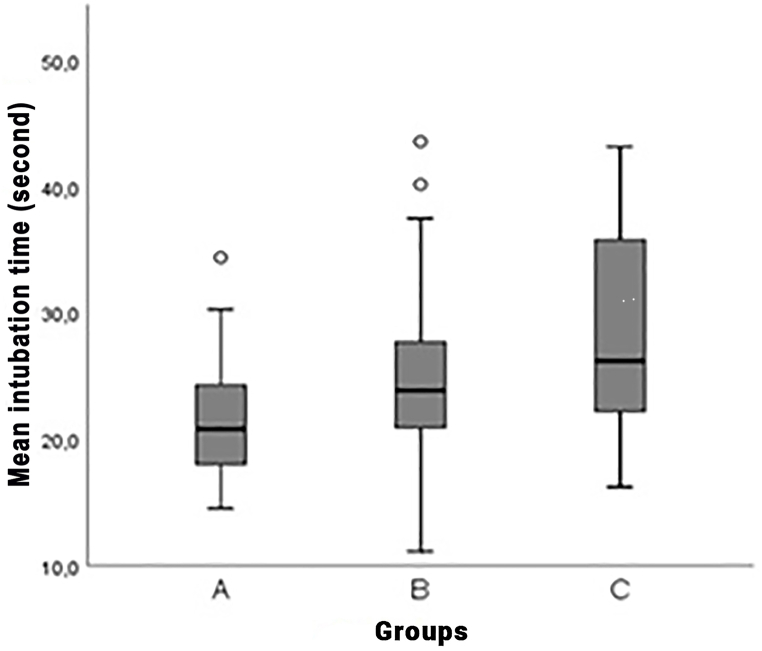
Distribution of mean intubation times in study groups.

Table 2 and Fig. 1 show the time distribution for participants to perform intubation. While all participants in Group A succeeded in the intubation procedure, one participant in Group B and two in Group C failed. Group C had the highest median intubation time (26.2 s), and there was a significant difference between Group A (20.8 s) and C in terms of median intubation time (p = 0.011). The average intubation times of the intubation performed by 21 different paramedic personnel on simulation manikins in all three groups are shown in Fig. 4.

**Table 2:** Distribution of the participants' intubation time.

	Groups		
	Group A	Group B	Group C
**Result**	Median (IQR)	Median (IQR)	Median (IQR)
Successful (sec)	20.8 (6.3)^a^	23.9 (6.8)	26.2 (15.0)^a^
Not successful (sec)	–	17.0 (−)	21.7 (−)

a There is a significant difference (*p* = 0.011).

## 5 Discussions

There are many studies in which PPEs used by healthcare personnel during the interventions to CBRN incidents cause delays or failures [[4], [5], [6], [7], [8], [9]]. For this reason, PPEs that minimize time loss should be preferred, especially in cases where an advanced airway is required. The present study revealed that full-face masks with air filters and panoramic visors used as PPEs prolong the time of intubation of healthcare personnel, and especially the use of masks with side air filters makes a significant difference in terms of time.

Medical personnel wearing CBRN-PPEs usually give first aid to the injured on the ground if they are in a hot area. Studies report that endotracheal intubation with a full-face mask on a manikin on the floor is more difficult than at waist level [4]. For this reason, a laryngeal mask airway (LMA) should be used on injured people on the floor in the contaminated area, and endotracheal intubation should be performed after taking them to a clean place [4]. Our study found that a side-mounted full-face mask with an air filter prolongs the intubation time. This is linked to the air filter on the left side because, during intubation of the manikin on the floor, the left arm of the paramedic has reduced maneuverability (Fig. 3a).

Since butyl rubber gloves used as personal protective equipment prevent fine motor movements, intraosseous intervention has been suggested for vascular access [5]. Especially in mass events with a large number of casualties, it has been argued that LMA for advanced airway and intraosseous intervention for vascular access would be more appropriate [6]. Another study conducted between anesthesiologists and non-anesthesiologists found that LMA was superior to endotracheal intubation [7]. If endotracheal intubation is to be performed in cases of mass injury, we recommend that a front air filter mask should be preferred in terms of time spent for intubation.

As we see in a study comparing video laryngoscope and Macintosh laryngoscope for endotracheal intubation under CBRN-PPEs, video laryngoscope is recommended [8].

Studies have generally suggested that the most convenient and quickest of medical interventions are under CBRN-PPEs. However, there are few studies on selecting CBRN-PPEs suitable for medical interventions. In the study of Brinker et al., in which they compared the convenience of binocular and panoramic visor masks during advanced life support interventions, they stated that masks with panoramic visors are more convenient [9]. Our study found that the front air filter masks would be better in terms of time and convenience for endotracheal intubation compared to the other types of panoramic type full-face masks. In this aspect, our study is one of the rare studies in the literature.

Group C had the highest median intubation time (26.2 s), and there was a significant difference between Group A (20.8 s) and Group C in terms of median intubation time (p = 0.011). There is a difference of 5.4 s between Group A and Group C. This difference can be seen as an insignificant time for routine endotracheal intubation. However, considering that there are many casualties in mass CBRN incidents and few medical personnel wearing CBRN-PPEs, this 5.4 s will become crucial. Appropriate equipment should be selected so professionals can perform medical intervention procedures quickly and correctly.

## 6 Limitations

There are two limitations of this study.

1 During the trials, only masks were worn by the healthcare personnel as CBRN-PPEs. For this reason, the negativities that may arise from other PPE, which should be used to intervene in a real CBRN casualty in a hot area, have been ignored.
2 The medical personnel were not stressed, as the trials were conducted under normal conditions, assuming that there was only one casualty without worrying about toxic gas. It was carried out under safe and secure conditions without any disturbance. In actual mass CBRN incidents, unfavorable conditions may affect the success of interventions due to the anxiety of medical personnel and the use of PPEs.

## 7 Conclusions

Using a front air filter mask with a panoramic visor during the intervention of CBRN casualties who need advanced airway may shorten the intubation time compared to side air filter masks. With further studies, these masks will become more ergonomic, easy and fast wearable products that allow better fine motor movements.

## Data availability statement

The data used in this study were directly collected during the course of simulations. No archived data were used.

## CRediT authorship contribution statement

**Orhan Delice:** Writing – review & editing, Visualization, Validation, Resources, Methodology, Investigation, Formal analysis, Data curation, Conceptualization. **Muhammet Özkul:** Writing – review & editing, Visualization, Validation, Methodology, Formal analysis, Conceptualization.

## Declaration of competing interest

The authors declare that they have no known competing financial interests or personal relationships that could have appeared to influence the work reported in this paper.
